# Iatrogenic wandering spleen causing gastric outlet obstruction and perforation

**DOI:** 10.1093/jscr/rjae088

**Published:** 2024-02-22

**Authors:** K R Lieb, D Beaulieu, M Dhir

**Affiliations:** School of Medicine, SUNY Upstate Medical University, Syracuse, NY 13210, United States; Department of Surgery, SUNY Upstate Medical University, Syracuse, NY 13210, United States; Division of Surgical Oncology, Department of Surgery, SUNY Upstate Medical University, Syracuse, NY 13210, United States

**Keywords:** case report, splenic ptosis, wandering spleen, adrenalectomy, complication

## Abstract

The phenomenon of a wandering spleen is rare with few published case reports. The cases published in the literature mainly result from acquired or congenital laxity of the spleen’s anchoring ligaments. Our case demonstrates an uncommon complication and possibly the first reported case of an iatrogenic wandering spleen. We present an interesting case of a 51-year-old female patient with congenital adrenal hyperplasia, fibromyalgia, and rheumatoid arthritis who underwent robotic-assisted left adrenalectomy for a 10-cm adrenal mass. Postoperatively, she developed uncontrolled pain and gastric distension due to spleen entrapment, leading to an open laparotomy and splenectomy with gastric perforation repair. She made an uneventful recovery. The rarity of iatrogenic wandering spleen as well as our patient’s complex medical history, including chronic steroid use, presented unique challenges in postoperative management. This case highlights the importance of thorough perioperative evaluation and careful surgical planning in patients with underlying conditions.

## Introduction

We present a rare case of iatrogenic wandering spleen following robotic-assisted left adrenalectomy in a female with congenital adrenal hyperplasia (CAH), fibromyalgia, and rheumatoid arthritis. To the best of our knowledge, the occurrence of an iatrogenic wandering spleen following robotic adrenalectomy has not been previously reported in the literature.

## Case report

We describe a 51-year-old female with a medical history notable for CAH (specifically 21a-hydroxylase deficiency), fibromyalgia, and rheumatoid arthritis. She presented in July 2023 with a symptomatic 10-cm left adrenal mass, biopsy-proven myelolipoma. This mass has been present since 2000. She underwent magnetic resonance imaging of the spine and evaluation by a pain team. No evidence of spinal nerve involvement could explain her pain. After treatment options were comprehensively discussed, she made an informed decision to proceed with surgical resection.

The patient underwent a robotic-assisted left adrenalectomy with a resection of the greater than 10-cm retroperitoneal mass. She received intraoperative stress dose steroids as well as perioperative endocrine consultation for CAH management. The adrenalectomy was uneventful. A modified right lateral decubitus position allowed for the medialization of both the spleen and pancreas, with the left crus of the diaphragm exposed medially and the cardia of the stomach carefully preserved. Employing a combination of medial to lateral and inferior to superior dissection techniques, the mass and adrenal gland were removed. At the end of the case, there was no entrapment of the bowel, and excellent hemostasis was ensured.

**Figure 1:**
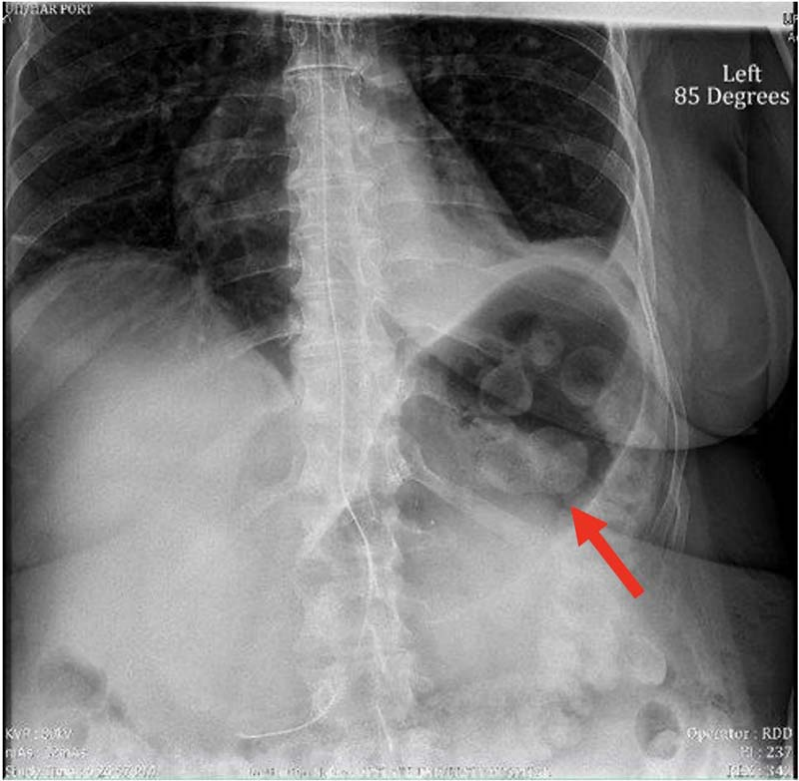
X-ray image of abdomen confirming persistent large gastric bubble despite adequately positioned NGT.

The patient was discharged on the second postoperative day following effective pain management and diet tolerance. However, she presented in the emergency department the following day with uncontrolled pain. Work-up revealed normal white blood cell count, absence of fever, and regular bowel function; she was admitted for pain control and IV hydration. She was not tolerating adequate oral intake. An abdominal X-ray was obtained showing a large gastric bubble, as seen in Fig. 1. The decision was made to place a nasogastric tube (NGT) for gastric decompression.

**Figure 2:**
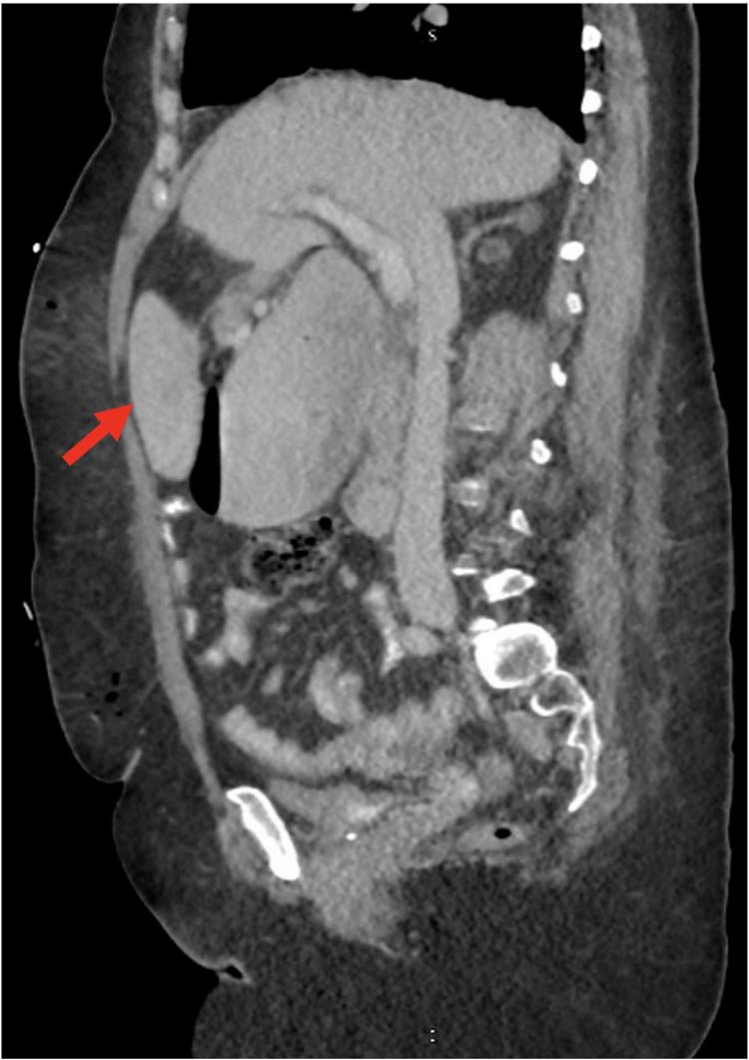
CT of the abdomen and pelvis (sagittal slice) showing gastric distention and the spleen positioned anteriorly.

**Figure 3:**
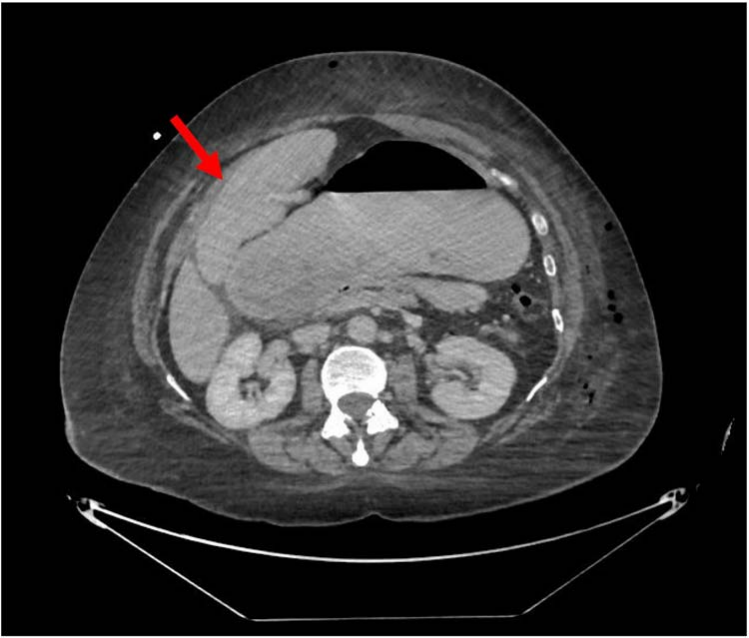
CT of the abdomen and pelvis (axial slice) showing gastric distention and the spleen positioned anteriorly in the right upper quadrant.

**Figure 4:**
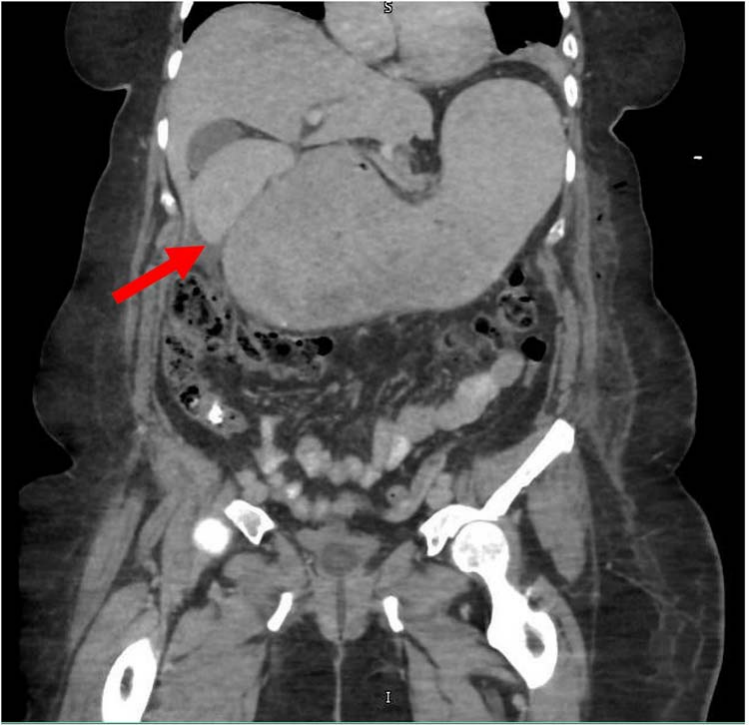
CT of the abdomen and pelvis (coronal slice) showing gastric distention and the spleen positioned in the right upper quadrant.

Despite the insertion of the NGT yielding 1500 cc output in 24 hours, the patient continued to experience severe abdominal pain. Urgent abdominal and pelvic computed tomography (CT) scans were taken, as seen in Figs 2–4. The CT scans showed gastric distension and the spleen positioned anteriorly in the right upper quadrant. Consequently, the decision was made to promptly bring the patient to the operating room for a diagnostic laparoscopy with plan to detorse the spleen.

Laparoscopy revealed that the spleen was entrapped between the falciform ligament and the stomach, most likely compressing the pylorus. An initial laparoscopic attempt was made to untwist the spleen, but there was deserosalization of the capsule, requiring the placement of a HandPort. As the spleen was enlarged, and could only be partially mobilized to the midline, the decision was made to convert to an open laparotomy and perform a splenectomy.

The spleen was mobilized, and a vascular load of stapler was used to staple the splenic hilum. The tail of the pancreas remained intact, and the spleen was extracted. Immediately beneath the spleen, a contained perforation of the stomach was identified. Using an open linear stapler, a stapled wedge resection was performed to excise the gastric wall defect, which was sent to pathology.

At this time, esophagogastroduodenoscopy was performed revealing an airtight closure, successful gastric decompression, and visualization of the duodenum. A drain was placed in the left upper abdomen, adjacent to the tail of the pancreas and stomach repair, and an NGT was positioned within the stomach. Her postoperative course was uneventful.

## Discussion

A wandering spleen, also known as splenic ptosis, is a rare condition characterized by the spleen’s abnormal migration throughout the abdominal cavity [1]. The abnormality or absence of its ligaments renders the spleen and its vascular pedicle mobile and subsequently susceptible to elongation and torsion [2, 3]. Instances of this condition manifest primarily as congenital abnormalities in children or as hormonal-mediated abdominal wall laxity in multiparous women, giving it a bimodal distribution between these two populations [4]. While conservative treatment was historically favored, the well-understood risks of splenic torsion have prompted surgical measures aimed at preventing this complication as comprehensively as possible [4]. These risks include splenomegaly, ischemic necrosis of the spleen, and rarely, left-sided portal hypertension [5, 6].

Published literature describing this phenomenon is sparse. Moore *et al.* [1] compiled seven reported cases of gastric outlet obstruction secondary to a wandering spleen and emphasized the need for further discourse surrounding this rare condition. They describe two adult patients treated with splenectomy and four younger patients treated with splenopexy. After converting to an exploratory laparotomy, we quickly decided on a splenectomy given the vascular congestion of the spleen secondary to the torsion and capsule deserosalization. While a splenopexy is generally the preferred option in uncomplicated wandering spleen cases, splenic torsion and other complications can mandate partial or total splenectomy [1].

Interestingly, none of the previously published wandering spleen cases are iatrogenic in origin. To our best knowledge, we describe the first case of iatrogenic wandering spleen following a robotic-assisted adrenalectomy. In our patient, the large size of the left adrenal mass and enlarged preoperative spleen required thorough mobilization of the spleen and tail of the pancreas to provide adequate access to the adrenal vein. These factors led to potential entrapment of the spleen under the falciform ligament, as well as potential torsion of the spleen with further enlargement compressing the pylorus, resulting in gastric outlet obstruction. Additionally, potential torsion along the short gastric vessels of the stomach could have led to the ischemic contained perforation of the stomach.

Our patient’s pre-existing conditions presented challenges in distinguishing between expected postoperative discomfort and potential complications following her recent adrenalectomy. This uncertainty, combined with a normal WBC count, absence of fever, and regular bowel function, initially led the medical team to attribute her pain to the recent surgery two days prior, delaying her diagnosis and potentially exacerbating her condition. Subsequent imaging became imperative, revealing a persistent large gastric bubble despite NGT decompression, and malposition of the spleen (Fig. 3). This prompted immediate preoperative measures to facilitate a diagnostic laparoscopy.

## Conflict of interest statement

The authors have no conflicts of interest to disclose.

## Funding

The authors have no relevant financial relationships or in-kind support to disclose.
